# The National Institutes of Health funding for clinical research applying machine learning techniques in 2017

**DOI:** 10.1038/s41746-020-0223-9

**Published:** 2020-01-31

**Authors:** Amarnath R. Annapureddy, Suveen Angraal, Cesar Caraballo, Alyssa Grimshaw, Chenxi Huang, Bobak J. Mortazavi, Harlan M. Krumholz

**Affiliations:** 1 Center for Outcomes Research and Evaluation, Yale-New Haven Hospital, New Haven, CT USA; 2 Section of Cardiovascular Medicine, Department of Internal Medicine, Yale School of Medicine, New Haven, CT USA; 3 Department of Internal Medicine, University of Missouri Kansas City School of Medicine, Kansas City, MO USA; 4 Harvey Cushing/John Hay Whitney Medical Library, Yale University, New Haven, CT USA; 5 Department of Computer Science & Engineering, Texas A&M University, College Station, TX USA; 6 Department of Health Policy and Management, Yale School of Public Health, New Haven, CT USA

**Keywords:** Outcomes research, Machine learning

## Abstract

Machine learning (ML) techniques have become ubiquitous and indispensable for solving intricate problems in most disciplines. To determine the extent of funding for clinical research projects applying ML techniques by the National Institutes of Health (NIH) in 2017, we searched the NIH Research Portfolio Online Reporting Tools Expenditures and Results (RePORTER) system using relevant keywords. We identified 535 projects, which together received a total of $264 million, accounting for 2% of the NIH extramural budget for clinical research.

## Introduction

Machine learning (ML) is the study of computer science and statistics that focuses on recognizing patterns and making inferences by analyzing large amounts of data, potentially with no explicit assumptions about these patterns. With the unprecedented availability of medical data from electronic health records, administrative claims, and registries, ML has the potential to transform clinical medicine. Many studies have highlighted the potentially transformative role of ML in medicine, including its use in disease stratification and diagnosis,^1–3^ to personalized risk prediction, and for imaging-based diagnostic purposes.^4^

Adequate funding is essential to explore the full potential of ML in clinical research and to develop products that could be implemented in real-world practice. To harness the potential of ML, the United States’ National Institutes of Health (NIH) has taken steps to increase the use of applications of ML in research by establishing programs, such as a program for artificial intelligence (AI), machine learning, and deep learning (https://www.nibib.nih.gov/research-funding/machine-learning), and by hosting workshops on using AI and ML to advance biomedical research (https://videocast.nih.gov/summary.asp?live=28053&bhcp=1). Yet, little is known about the total number or the total dollar amount that is allotted to clinical research projects that apply ML techniques. Moreover, there is little knowledge about which funding agencies of the NIH fund the most clinical research applying ML, and which grant types are funded, such as research grants (R series) and career development grants (K series). This knowledge could guide investigators and academic medical centers to compile their applications competitively for the appropriate NIH centers to increase the probability of being funded. Additionally, such understanding could reveal gaps in the types of ML studies being funded, which might inform decisions about future funding. Accordingly, we sought to describe and characterize the recipients of NIH funding for ML in 2017.

## Results

### Baseline characteristics

Using selected keywords, we identified 1960 projects from the NIH Research Portfolio Online Reporting Tools Expenditures and Results (RePORTER) system, of which 535 met our inclusion criteria. Together, these projects received $264,941,309 in funding, accounting for 2% of the NIH extramural budget for clinical research for fiscal year (FY) 2017 ($12,695 million) (https://report.nih.gov/categorical_spending.aspx). The median and maximum amount per project was $347,944 (Interquartile range, $187,582–586,327) and $12,560,000, respectively (Supplementary Table 1). Of the 535 grants, 15 were subprojects with duplicate project numbers, and 13 received awards from more than one agency of the NIH.

### Funding by NIH agency and mechanism

The projects included in this study were funded by 26 NIH agencies. Four agencies contributed nearly half of the total funding awarded: the National Caner Institute (15%), National Institute of Mental Health (11%), National Institute on Aging (10%), and National Heart, Lung, and Blood Institute (9%) (Table 1). Grants were awarded by 54 different funding mechanisms (Table 2). Of these, investigator-initiated R01 research grants received the highest amount of funding (39%) followed by U54 and U24 grants (7% of the total amount of funding for each type). Among the nine application types, those for non-competing continuation (type 5) received $138,151,114, accounting for 52% of the total amount, while funding for new applications (type 1) represented 40% of the total amount (Supplementary Table 2). Almost half of the 535 projects received funding for 2–5 years, while one third received funding for 1 year (Supplementary Table 3). Furthermore, 151 projects were registered at ClinicalTrials.gov.

**Table 1:** Machine learning grants awarded by an institute of the National Institutes of Health (NIH).

Institute/center code	Total number of grants	Proportion of total grants (%)	Total grant amount ($)	Proportion to total value of grants (%)
National Cancer Institute (NCI)	66	11.7	39,298,859	14.8
National Institute of Mental Health (NIMH)	57	10.1	28,927,076	10.9
National Institute on Aging (NIA)	45	8.0	26,303,801	9.9
National Heart, Lung, and Blood Institute (NHLBI)	44	7.8	22,602,244	8.5
National Institute of General Medical Sciences (NIGMS)	40	7.1	18,585,533	7.0
Office of the Director, NIH (OD)	30	5.3	17,103,011	6.5
National Institute on Neurological Disorders and Stroke (NINDS)	37	6.6	16,830,246	6.4
National Library of Medicine (NLM)	39	6.9	14,473,321	5.5
National Institute of Biomedical Imaging and Bioengineering (NIBIB)	30	5.3	12,640,025	4.8
National Institute on Drug Abuse (NIDA)	26	4.6	10,917,625	4.1
National Institute of Allergy and Infectious Diseases (NIAID)	20	3.6	10,480,116	4.0
National Institute of Child Health and Human Development (NICHD)	28	5.0	8,047,239	3.0
National Institute on Deafness and Other Communication Disorders (NIDCD)	15	2.7	7,658,753	2.9
National Institute of Diabetes and Digestive and Kidney Diseases (NIDDK)	14	2.5	5,112,183	1.9
Center for Information Technology (CIT)	5	0.9	5,038,047	1.9
National Center for Advancing Translational Sciences (NCATS)	5	0.9	4,624,239	1.7
National Institute of Nursing Research (NINR)	8	1.4	2,548,731	1.0
National Institute on Alcohol Abuse and Alcoholism (NIAAA)	9	1.6	2,471,394	0.9
National Human Genome Research Institute (NHGRI)	8	1.4	2,424,273	0.9
National Eye Institute (NEI)	7	1.2	2,296,364	0.9
National Institute of Environmental Health Sciences (NIEHS)	11	2.0	2,025,184	0.8
National Center for Complementary and Integrative Health (NCCIH)	4	0.7	1,581,038	0.6
National Institute on Minority Health and Health Disparities (NIMHD)	3	0.5	1,271,018	0.5
National Institute of Arthritis and Musculoskeletal and Skin Diseases (NIAMS)	5	0.9	1,260,046	0.5
National Institute of Dental and Craniofacial Research (NIDCR)	3	0.5	226,522	0.1
Fogarty International Center (FIC)	2	0.4	194,421	0.1

**Table 2:** The NIH grants by funding mechanism.

Activity code	Total number of grants	Proportion of total number of grants (%)	Total grant amount ($)	Proportion to total value of grants (%)
R01	208	38.9	108,606,595	41.0
U54	20	3.7	19,550,967	7.4
U24	11	2.1	18,959,934	7.2
U01	17	3.2	16,548,928	6.2
P50	19	3.6	11,192,516	4.2
R44	15	2.8	10,234,849	3.9
R56	5	0.9	9,763,193	3.7
ZIA	17	3.2	9,210,836	3.5
R21	39	7.3	8,201,484	3.1
OT2	1	0.2	5,360,833	2.0
P30	16	3.0	3,862,098	1.5
P01	8	1.5	3,649,816	1.4
R43	18	3.4	3,476,503	1.3
K01	20	3.7	3,376,740	1.3
ZIH	2	0.4	3,123,589	1.2
DP2	1	0.2	2,542,500	1.0

Of the institutions that received grants, the ones that received most of the funding (25% of the total amount) were Stanford University (8.6%), University of Pittsburgh (4.9%), University of North Carolina (4.7%), University of Wisconsin-Madison (3.3%), Indiana University-Purdue University at Indianapolis (3.1%), and University of California Los Angeles (2.7%). Overall, 77 out of 207 (37%) institutions and 49 out of 469 (10%) principal investigators received multiple grants.

## Discussion

Our study provides a snapshot of federal funding for clinical research projects using ML techniques in the United States. In FY 2017, 535 projects received $264 million, accounting for a relatively small proportion of the NIH budget for clinical research. This study highlights the small proportion of NIH funding that is allotted for clinical research projects applying ML, techniques that have immense potential to transform health care and add to the ongoing debate about NIH funding priorities.

ML is applied in various disciplines of medicine, yet 12 out of 27 agencies of the NIH each funded fewer than 10 clinical research projects applying ML. Therefore, these findings could represent opportunities for increasing future funding for ML by these NIH centers. Consistent with the general trend of NIH funding patterns, investigator-initiated projects were comparatively well funded through the R01 mechanism, constituting more than one third of the total number of grants.^5^ In contrast, funding mechanisms to train the next generation of scientists, including fellowships (F series), research training (T series), and career development grants (K series), which represent less than one fifth of the total number of grants, received less support. Training and career development awards in ML are critical for fostering interest among early-career scientists and/or physicians by providing protected time, and for ensuring successful transformation to R awards; studies have reported the 10-year K-to-R successful conversion rate to be between 30–40%.^6^

Another important finding is the concentration of funding in a small number of research institutions. It may be useful for the NIH to consider how best to increase the capacity of more institutions to participate in producing knowledge for the next generation of medicine and health care. There may be mechanisms to strengthen the expertize across a broader range of institutions.

The NIH has been focused on building infrastructure for ML and has endorsed its importance. In 2013, the NIH launched the Big Data to Knowledge (BD2K) program to support research and development of innovative and transformative approaches and to maximize and accelerate the integration of big data and data science into biomedical research.^7^ Recently, the NIH launched the All of Us Research Program (https://allofus.nih.gov) as part of its precision medicine initiative, with the objective to collect environmental, clinical, imaging, and laboratory data from 1 million or more people, with plans to make the data publicly available for investigators. In addition, the NIH mandated that all clinical trials funded by the NIH should be made publicly available. Dr. Francis Collins, director of the NIH, stated that “the advent of artificial intelligence and machine learning, big data, cloud computing, and robotics may represent the Fourth Industrial Revolution” (https://datascience.nih.gov/sites/default/files/AI_workshop_report_summary_01-16-19_508.pdf). Our study suggests that in clinical research, however, NIH funding for ML remains modest.

The study should be interpreted in the context of some limitations. We used a systematic approach to identify projects that applied ML techniques to population and clinical research. Because RePORTER makes available only the abstract section of a grant, some qualified projects with insufficient information might have been excluded. More sharing of proposals would benefit the field. Nevertheless, previous studies followed an approach similar to ours in characterizing data from RePORTER.^5,8^ In addition, we do not have access to information about unfunded NIH applications. Hence, we cannot measure the proportion of projects using ML that received funding. We focused on modeling techniques implemented regardless of the learning tasks, because learning tasks, such as supervised learning, can also be implemented in traditional regression models. Additionally, we did not include linear regression, logistic regression, or regressions with regularizations (e.g., a ridge regression that uses the L2 penalization) for models. However, this approach is consistent with the manner in which the majority of published papers self-identify as having used ML approaches for model-building.^9^

In conclusion, our study provides information on contemporary funding for clinical research projects that apply ML techniques, which we found represents a small percentage of NIH-funded research. Almost all agencies of the NIH support projects that use ML through different grant mechanisms, with training and career development grants receiving the least support. Therefore, to harness advances in ML and computational power, more NIH-sponsored support for clinical research using these techniques, especially for training future scientists, is necessary.

## Methods

### Study sample and search strategy

We searched for all NIH-funded studies for the 2017 FY (October 2016–September 2017) by using the RePORTER system (https://projectreporter.nih.gov/reporter.cfm), a publicly accessible tool that contains comprehensive information about research projects funded by the NIH. We specifically sought out information on clinical research projects using ML. For this study, to determine if ML was utilized, we defined ML by the modeling techniques, rather than the specific learning tasks. The algorithms, namely Trees (e.g., random forest), Support vector Machine, and neural networks, are modeling approaches that can be used with different types of learning tasks, including supervised, unsupervised, semi-supervised, and reinforcement learning. We searched project titles, project abstracts, and project terms using the following keywords:^10,11^ “artificial intelligence,” “Bayesian learning,” “boosting,” “gradient boosting,” “computational intelligence,” “computer reasoning,” “deep learning,” “machine intelligence,” “machine learning,” “naive Bayes,” “neural network,” “neural networks,” “networks analysis,” “natural language processing,” “support vector machines,” “random forest,” “computer vision systems,” and “deep networks.” Alternative versions of these keywords have been tested to ascertain if abstracts could be identified, and those found useful have been included in the final list of keywords. We restricted our search to centers affiliated with the NIH and excluded projects funded by the Centers for Disease Control and Prevention, the Food and Drug Administration, the Agency for Healthcare Research and Quality, the Health Resources and Services Administration, and the Department of Veterans Affairs because RePORTER did not have comprehensive data on these grants.

For this study, we were primarily interested in the funding for projects using population health data, and applied inclusion and exclusion criteria to obtain the grants related to those projects. We included projects that used population or clinical research data and that explicitly mentioned the use of one of the above-named ML techniques in their abstract. We considered a project to be population or clinical research if it contained data related to demographics, imaging, anatomopathologics, and biomarkers, or if it had direct involvement of humans such as social science research. We excluded projects that used ML only on basic science research to gain biological insights into mechanisms of disease (e.g., ML approaches for electrophysiological cell classification); those that used ML on solely biological data, such as genomic, proteomic, or RNA sequencing data without incorporating clinical or demographic data (e.g., a study to use state-of-the-art methods from the fields of ML, statistics, or natural language processing to improve the ability to make sense of large tandem mass spectrometry data sets); and those that used ML only on data from animal experiments.

### Data collection and analysis

We used Covidence (Covidence, Melbourne, Australia), an online software tool to screen the project abstracts (https://www.covidence.org/home*)*. Two investigators (SA and CC) independently screened each abstract for inclusion and exclusion criteria and agreement was required to include or exclude a project. When there was disagreement, a third investigator (ARA) resolved the conflict. Numbers and titles of the projects included in the final analysis are listed in the Supplementary Table 4.

We described total dollar amounts, number of grants, and median dollar amount per grant. Additionally, we calculated proportion of total dollar amounts and total number of grants by funding agency of the NIH (e.g., NCI, NAI, and NIGMS), application type (new application, continuity of application), funding mechanism (e.g., R01, U01, and F32), and number of supported years. Stata Version 15.0 (StataCorp, College Station, Texas) and Microsoft Excel^®^ were used for analysis. Since the data used were publicly available and did not contain patient information, the study was exempted from review by the Yale University Institutional Review Board.

### Reporting summary

Further information on research design is available in the Nature Research Reporting Summary linked to this article.

## Supplementary information

Supplementary Tables

Reporting Summary

## Data Availability

The data for the current study were generated from the NIH's Research Portfolio Online Reporting Tools Expenditures and Results (RePORTER) system, which are publicly available at https://projectreporter.nih.gov/reporter.cfm. The numbers and titles of the NIH projects included in the final analysis are listed in Supplementary Table 4.
