# Supplementary material for: The National Institutes of Health funding for clinical research applying machine learning techniques in 2017
Source: NPJ Digit Med. 2020 Jan 31;3:13. doi: 10.1038/s41746-020-0223-9 (PMC6994580; doi:10.1038/s41746-020-0223-9)
Supplement: Supplementary file 1 — Supplementary Tables [file 41746_2020_223_MOESM1_ESM.pdf]

Supplemental tables

Supplementary Table 1. The NIH Funding for Clinical Research Applying Machine Learning in 2017

Supplementary Table 2. NIH funding by application type

Supplementary Table 3. NIH grants by supporting year

Supplementary Table 4. Project numbers and Project titles of NIH grants

**Supplementary Table 1. The NIH Funding for Clinical Research Applying Machine Learning in 2017**	
Total number of projects screened	1964
Total number of projects excluded	1423
Total number of unique project identification numbers	520
Total number of projects	535
Total amount of funding, $	264,941,309
Median (IQR), $	341,475 (183,049–581,327)
Mean (SD), $	495,217 (810,048)
Maximum amount received by a single grant, $	12,560,000
Number of Principal Investigators	470
Number of institutions	207
Number of NIH agencies	27
Number of grant mechanisms	54
NIH total budget for FY 2017, $	33,136,000,000
NIH extramural budget for FY 2017, $	29,622,000,000
Number of projects registered in ClinicalTrials.gov	151

IQR, interquartile range; SD, standard deviation; FY, fiscal year; NIH, National Institutes of Health

**Supplementary Table 2. NIH funding by application type**				
**Application type**	**Number of grants**	**Proportion of total number of grants**	**Total grant amount ($)**	**Proportion of total value**
Noncompeting continuation (5)	292	54.8%	138,151,114	52.2%
New application (1)	187	35.1%	106,077,096	40.1%
Renewal (2)	29	5.4%	13,862,777	5.2%
Revision (3)	18	3.4%	3,457,700	1.3%
Change of grantee (7)	5	0.9%	2,609,456	1.0%
Change of institute (9)	1	0.2%	359,872	0.1%
Extension (4)	1	0.2%	248,807	0.1%

Supplementary Table 3. NIH grants by supporting year

Support year	Total number of grants	Proportion of total number of grants	Total grant amount ($)	Proportion of total value of grants
1 yr	176	32.9%	96,728,108	36.5%
2–5 yrs	264	49.3%	124,454,116	47.0%
6–10 yrs	39	7.3%	20,696,923	7.8%
>10 yrs	56	10.5%	23,062,162	8.7%

Supplementary Table 4. Project numbers and Project titles of NIH grants

	Project number	Project Title
1	1DP2MD012722-01	Machine Learning For Health Outcomes And Quality Of Care In Low-Income Populations
2	1F30HL136001-01A1	Perinatal Origins Of Asthma
3	1F30MH115584-01	Genome Clustering For Clinical Subtype Detection In Autism
4	1F31CA214029-01	Genotype And Histological Phenotype Relationships In Cancer; With Automated Therapy Optimization.
5	1F31DA043328-01A1	Predicting Stimulant Use Relapse Using Neuroimaging Techniques
6	1F31HD091967-01	Testing The Predictive Power Of Structural Neuroimaging In The Estimation Of Individuals' Reading And Attentional Abilities
7	1F32HD091945-01	Understanding Severe Maternal Morbidity: Predictors; Trends; And Disparities
8	1F99NS105210-01	A Hybrid Neural-Machine Interface For Volitional Control Of A Powered Lower Limb Prosthesis
9	1K01DA041620-01A1	Using Administrative And Clinical Data To Detect Drug Use And HIV Risk In Foster Care
10	1K01ES028055-01	Using Clinical Data To Identify FDA-Approved Drugs For Cancer Prevention And Therapeutic Repurposing
11	1K01HD091283-01	Big Data Neuroimaging To Predict Motor Behavior After Stroke
12	1K01HD093863-01	Big Data And Network Analysis Of Children's Health
13	1K01HL135452-01	The Effect Of Insufficient Sleep On Cardiovascular Disease Disparity Between Blacks And Whites
14	1K01HL136687-01	Data-Driven Identification Of The Acute Respiratory Distress Syndrome
15	1K01HL137557-01	Prediction Of Heart Failure In HIV-Infected Individuals
16	1K01LM012529-01A1	Improved Disease Stratification Using Electronic Health Records
17	1K02DA043063-01	Classifying Addictions Using Machine Learning Analysis Of Multidimensional Data
18	1K02NS104207-01	Quantitative Rehabilitation After Stroke
19	1K08EB022631-01A1	Identifying Opioid Response Phenotypes In Low Back Pain Electronic Health Data
20	1K08HL136850-01	Improving Cardiovascular Drug Safety With Automated Bleeding Classification
21	1K08HL136928-01	Multi-Omic Subtyping Of Chronic Obstructive Pulmonary Disease
22	1K24DC016312-01	Prolonging Functional Speech In Persons With Amyotrophic Lateral Sclerosis: A Real-Time Virtual Vocal Tract
23	1K99AR070902-01A1	Multidimensional Mri-Based Big Data Analytics To Study Osteoarthritis
24	1K99EB023279-01A1	Spectroscopic Photoacoustic Molecular Imaging For Breast Lesion Characterization
25	1K99ES027022-01A1	A Data Science Approach To Air Toxics And Children's Environmental Health
26	1K99ES027511-01A1	Air Pollution Exposures And Children's Health: Mediation And Interaction In A Counterfactual Framework
27	1K99HG009680-01	Beckon - Block Estimate Chain: Creating Knowledge On Demand & Protecting Privacy
28	1K99MH111807-01A1	Estimating Population Effects In Mental Health Research Using Meta-Analysis

29	1OT2OD025286-01	All Of Us; Wisconsin
30	1P01AI131374-01	Core B: Bioinformatics/Biostatistics Core
31	1P01AI131374-01	Project 3: Identifying Plasma Biomarkers Predicting Time To HIV Rebound After Treatment Interruption
32	1P42ES027704-01	Data Science Core
33	1P50AR070590-01A1	Core 2: Applied Meta'omics Core
34	1P50CA211024-01A1	Computational/Biostatistical Core
35	1R01AG053949-01A1	Advanced Machine Learning Algorithms That Integrate Genomewide; Longitudinal Mri And Demographic Data To Predict Future Cognitive Decline Toward Dementia
36	1R01AG054523-01A1	Blood Base Bioenergetic Profiling: A Novel Approach For Identifying Alzheimer's Disease Risk And Pathology
37	1R01AG054628-01A1	Evaluating Longitudinal Changes In The Human Structural Connectome In Relation To Cognitive Aging
38	1R01AG055121-01A1	Predict-Adftd: Multimodal Imaging Prediction Of Ad/Ftd And Differential Diagnosis
39	1R01AG055132-01	Using Connectomics To Characterize Risk For Alzheimer's Disease
40	1R01AG055337-01A1	Precede: Presurgical Cognitive Evaluation Via Digital Clockfacedrawing
41	1R01AG056287-01	The Phenotypic Landscape Of Cognitive Decline As Revealed By Next-Generation Multiplexed Ion Beam Imaging
42	1R01AG056331-01	Sleep Health Profiles And Mortality Risk In Older Adults: A Multi-Cohort Application Of Novel Statistical Methods
43	1R01AG057915-01	Miriad - Multiplexed Imaging Of Resilience In Alzheimers Disease
44	1R01AI127203-01A1	Big Data Analytics Of Hiv Treatment Gaps In South Carolina: Identification And Prediction
45	1R01AI127250-01A1	Big Data Analysis Of Hiv Risk And Epidemiology In Sub-Saharan Africa
46	1R01AI130460-01	Dynamic Learning For Post-Vaccine Event Prediction Using Temporal Information In Vaers
47	1R01AI132030-01	Mining Real-Time Social Media Big Data To Monitor Hiv: Development And Ethical Issues
48	1R01CA203861-01A1	Augmented Neurosurgical Navigation Software Using Resting State Mri
49	1R01CA207375-01A1	The Effectiveness; Safety; And Costs Of Guideline-Concordant Lung Nodule Care
50	1R01CA211224-01A1	Devaluing Energy-Dense Foods For Cancer Control: Translational Neuroscience
51	1R01CA215318-01	Calibrating Free-Living Physical Activity Characteristics Across Functionally-Limited Populations Using Machine-Learned Accelerometer Approaches
52	1R01CA218144-01	Brain Cancer Radio-Pathomics For Predicting Heterogeneous Cytology
53	1R01DA042988-01A1	Effects Of Prenatal Cocaine On Early Brain Functional Connectivity And Behavior
54	1R01DA043690-01	Meta-Analysis And Machine Learning: Towards Neuromarkers Of Craving And Relapse
55	1R01DA044170-01	Using Existing Data To Understand And Ameliorate Risk In Opioid Agonist Therapy
56	1R01DA044985-01	Using Machine Learning To Predict Problematic Prescription Opioid Use And Opioid Overdose
57	1R01DK110541-01A1	Developing And Validating Prognostic Metabolomic Signatures Of Diabetic Kidney Disease
58	1R01DK113111-01	Consortium For Radiologic Imaging Studies Of Polycystic Kidney Disease (Crisp) Iv: Prognosis For End-Stage Renal Disease And Biomarker Validation

59	1R01DK113269-01	Development And Validation Of An Automated Measurement Of Child Screen Media Use: Flash
60	1R01EB023942-01	Deep Radiomic Decision Support System For Colorectal Cancer
61	1R01EB023943-01	Enabling Technology For Safe Robot-Assisted Surgical Micromanipulation
62	1R01EB025020-01	Qubbd: Deep Poisson Methods For Biomedical Time-To-Event And Longitude Data
63	1R01EB025024-01	Qubbd: Statistical & Visualization Methods For Pghd To Enable Precision Medicine
64	1R01ES027747-01	Air Particulate; Metals; And Cognitive Performance In An Aging Cohort- Roles Of Circulating Extracellular Vesicles And Non-Coding Rnas
65	1R01GM120624-01A1	Computational Studies Of Virus-Host Interactions Using Metagenomics Data And Applications
66	1R01GM123193-01	Sepsis Early Prediction And Subphenotype Illumination Study (Sepsis)
67	1R01HD078532-01A1	Developing Inhibitory Control
68	1R01HD087363-01A1	Ped Screen: Pediatric Sepsis Ehr Registry; Clinical Outcomes; And Predictive Model
69	1R01HD091179-01	Neonatal Endotracheal Intubation: Enhancing Training Through Computer Simulation And Automated Evaluation
70	1R01HD092239-01	Instrumental Screening For Dysphagia By Combining High-Resolution Cervical Auscultation With Advanced Data Analysis Tools To Identify Silent Dysphagia And Silent Aspiration
71	1R01HD092331-01	Externalizing Outcomes In High Risk Youth
72	1R01HL133786-01A1	Exploring Statin Pleiotropic Effects Within A Very Large Ehr Cohort
73	1R01HL134673-01	Phora: A Clinical Decision Support Tool For Patients With Pulmonary Arterial Hypertension
74	1R01HL135219-01	Community Surveillance Of Coronary Heart Disease
75	1R01HL135557-01A1	Integrated Analysis Of Coronary Anatomy And Biology With 18f-Fluoride Pet And Ct Angiography
76	1R01HL136660-01	Automated Detection And Prediction Of Atrial Fibrillation During Sepsis
77	1R01HL137763-01	Magnetic Resonance Imaging Based Calf Muscle Perfusion To Assess Patients With Symptomatic Peripheral Artery Disease
78	1R01LM012535-01	Integrating Neuroimaging; Multi-Omics; And Clinical Data In Complex Disease
79	1R01LM012734-01	Mechanistic Machine Learning
80	1R01LM012815-01	Improving Patient And Caregiver Engagement Through The Application Of Data Science Methods To Audio Recorded Clinic Visits Stored In Personal Health Libraries
81	1R01MH104030-01A1	The New Tics Study: A Novel Approach To Pathophysiology And Cause Of Tic Disorders
82	1R01MH110453-01A1	Characterizing Trauma Outcomes: From Pre-Trauma Risk To Post-Trauma Sequelae
83	1R01MH111610-01A1	Lifesense: Transforming Behavioral Assessment Of Depression Using Personal Sensing Technology
84	1R01MH112070-01A1	Mapping Heterogeneity Of Neuroanatomical Imaging Signatures Of Psychosis Via Pattern Analysis
85	1R01MH113250-01	Identifying Reproducible Brain Signatures Of Obsessive-Compulsive Profiles
86	1R01MH113406-01	Machine Learning To Distinguish Hand From Alzheimer's Disease In Hiv Over Age 60
87	1R01MH113570-01A1	The Preterm Behavioral Phenotype: Trajectories Of Psychopathology & Changes In Cerebral Connectivity

88	1R01NR016732-01A1	A Clinician-In-The-Loop Smart Home To Support Health Monitoring And Intervention For Chronic Conditions
89	1R01NR016941-01	Communicating Narrative Concerns Entered By Rns (Concern)
90	1R01NS100849-01A1	Mid-Frontal Delta/Theta Rhythms And Cognitive Control In Pd
91	1R01NS102233-01	Enabling Comparative Effectiveness Research In Silent Brain Infarction Through Natural Language Processing And Big Data
92	1R03DE026513-01A1	Craniofacial Microsmia: Facial Expression From Ages 1 To 3 Years
93	1R13NS101927-01A1	Ispw8 Conference: Designing The Next Generation Of Closed Loop Seizure Control
94	1R21AA025193-01A1	A Machine Learning Approach For Inferring Alcohol Intoxication Levels From Gait Data
95	1R21AG055749-01	Automated Assessment Of Cognitive Tests For Detecting Mild Cognitive Impairment
96	1R21AR072263-01	Algorithms To Identify Systemic Lupus From Electronic Health Record Data
97	1R21CA212744-01	Characterization Of Thyroid Nodules By Quantitative Ultrasound
98	1R21CA219229-01	Using Big Data To Estimate The Effects Of Complex Cost-Sharing Rules On Colorectal Cancer Screening And Patient Health
99	1R21DA043171-01	Functional Dissection Of Prenatal Drug Effects On The Developing Brain And Behavior
100	1R21EB024025-01	Deep Radiomic Colon Cleansing For Laxative-Free Ct Colonography
101	1R21EY027945-01	Machine Learning Methods For Detecting Disease-Related Functional And Structural Change In Glaucoma
102	1R21HD087749-01A1	Vaccine Beliefs And Decision Making
103	1R21HD091500-01	Evaluation Of Machine Learning To Mobilize Detection And Therapy Of Developmental Delay In Children
104	1R21HD092243-01	A Functional Upper Limb Training And Assessment Tool To Enhance Efficacy And Scalability Of Rehabilitation In Ecological Environments
105	1R21LM012395-01A1	Deconvolution Of Epigenomic Data To Characterize Cellular Subpopulations
106	1R21LM012618-01	Bayesian Generative Methods For Extracting And Modeling Relations In Ehr Narratives
107	1R21MH113870-01	Naturalistic Data Collection In The Smartplayroom
108	1R21NS100244-01A1	Development Of Automated Web-Based Spectroscopic Mri Clinical Interface
109	1R21NS102828-01	Deep Learning For Connectomics
110	1R25EB024327-01	Development Of An Online Course Suite In Tools For Analysis Of Sensor-Based Behavioral Health Data (Aha!)
111	1R25MH112480-01	Summer Institute In Neuroimaging And Data Science
112	1R41AA025297-01A1	Patient Engagement Alcohol Risk Reduction System (Pearrs)
113	1R41AG056184-01	Telehealth Intervention For Improved Blood Pressure Control With Targeted Incentives: Using New Technologies And Insights From Behavioral Economics To Increase The Effectiveness Of Behavioral Change
114	1R42DA043977-01	Advancing A Novel Portable Detection Method For Cannabis Intoxication
115	1R43AA026492-01	Using The Lumme System To Improve Real-Time Blood Alcohol Detection
116	1R43AG057257-01	Augmented Reality System For The Education Of Clinical Caregivers Of Older Adults
117	1R43AG058354-01	Real-Time Video Monitoring Of Falls In Memory-Care Facilities For Individuals With Alzheimer's And Related Dementias

118	1R43AG058480-01	Predictive Analytics For Alzheimer's Disease Dementia
119	1R43AR073114-01	Optimizing Electrical Impedance Myography Outcomes Through Data Mining
120	1R43DA044062-01	Leveraging Predictive Analytics Within Social Networks To Maximize Drug And Alcohol Treatment Efficacy And Relapse Prevention
121	1R43DC016251-01A1	User-Driven Fitting Of Hearing Aids And Other Assistive Hearing Devices
122	1R43EB024299-01	Improving Access To Vision Correction For Health Disparity Populations With The Quicksee: An Accurate; Low-Cost; Easy-To-Use Objective Refractor
123	1R43GM122154-01	Rule-Based Semantics And Big Data Based Methods For Effective Clinical Decision Support (Cds): A Pediatric Severe Sepsis Case Study Using Icu Data
124	1R43GM122196-01	Centralized Assay Datasets For Modelling Support Of Small Drug Discovery Organizations
125	1R43GM123851-01	Development Of Deep Learning Models For Biomarker Identification And Classification
126	1R43HL135909-01A1	Rule Based Semantics And Big Data Based Methods For Early Identification Of Patients At Risk Of Acute Respiratory Distress Syndrome (Ards)
127	1R43LM012798-01	Enabling Value-Based Healthcare Through Automating Risk Assessment For Episode-Based Care
128	1R43MH113408-01	Evaluating The Comparative Validity And Reliability Of Seras: A Decision Support Tool For Assessing Near Term Risk Of Suicide In Emergency Departments
129	1R43MH114763-01	Detecting And Monitoring Tardive Dyskinesia To Improve Patient Outcomes
130	1R43NR015945-01A1	Autonomous System Supporting Patient-Specific Transfer And Discharge Decisions
131	1R43TR002047-01	Software Platform To Stratify Patients For Treatment Arm Randomization In Human Clinical Trials Using Patient-Level Predictive Models
132	1R44AG054256-01A1	Characterizing Alzheimer's Disease With Inspecds: Integrated Neurocognitive And Sleep-Behavior Profiler For The Endophenotypic Classification Of Dementia Subtypes
133	1R44DA044929-01A1	The Blackfynn Platform For Rapid Data Integration And Collaboration
134	1R44GM119858-01A1	Adapting The Berkeley Big Data Analytics Stack To Genomics And Health
135	1R44GM125438-01	Clinical Resting State Fmri Software For Surgical Planning
136	1R44HD093467-01	Automatic Positioning Of Communication Devices And Other Essential Equipment For People With Mobility Restrictions
137	1R44HL132622-01A1	Tailored Drug Titration Through Artificial Intelligence
138	1R44MH108177-01A1	Web Software To Develop An Rdoc-Compatible Adaptive Diagnostic Nosology; The Sid-5
139	1R56AG055728-01	Integrated Model Of Palliative And Primary Care In Seriously Ill Older Adults
140	1R56AG057195-01	Early Onset Alzheimer's Disease Consortium
141	1R56HL135425-01A1	Mhealth For Heart Failure: Predictive Models Of Readmission Risk And Self-Care Using Consumer Activity Trackers
142	1R56HL138415-01	Interpretable Deep Learning Model For Longitudinal Electronic Health Records And Applications To Heart Failure Prediction
143	1R56NS096064-01A1	Machine Learning-Based Control Of Functional Electrical Stimulation
144	1S10OD021644-01A1	From Genomics To Natural Language Processing: A Protected Environment For Research Computing In The Health Science
145	1T15LM012500-01	An Interdisciplinary Program For Advanced Training In Health Data Analytics
146	1U01CA214411-01A1	Engineering Personalized Micro-Tumor Ecosystems

147	1U01HL137159-01	Systems Level Causal Discovery In Heterogeneous Topmed Data
148	1U01LM012675-01	Crowd-Assisted Deep Learning (Cradle) Digital Curation To Translate Big Data Into Precision Medicine
149	1U01MH109985-01A1	Mapping Connectomes For Disordered Emotional States
150	1U01TR002062-01	Open Health Natural Language Processing Collaboratory
151	1U19AI128949-01	Data Management And Analysis Core
152	1U19AI129910-01	The Innate Immune Response As A Therapeutic Target For Cutaneous Leishmaniasis
153	1U19CA203654-01A1	Core B: Biostatistics Core
154	1U19NS104649-01	Data Science Core
155	1U19NS104653-01	Data Science Core
156	1U24CA224309-01	Immune Monitoring And Analysis Of Cancer At Stanford (Imacs)
157	1U24DK116204-01	Illuminating Function Of The Understudied Druggable Kinome
158	1U24HL138998-01	Data; Modeling;And Coordination Center For Precise Network
159	1U54CA209891-01A1	Using Networks To Seed Hierarchical Whole-Cell Models Of Cancer
160	1U54CA224019-01	Impact Of Leukemia Microenvironment On Response To Targeted Therapies In Aml
161	1U54HG009824-01	The Nasopharyngeal Microbiota Of African Children And Lower Respiratory Tract Infection
162	1U54NS105539-01	Topological Mapping Of Immune; Microbiota; Metabolomic And Clinical Phenotypes To Reveal Me/Cfs Disease Mechanisms - Clinical Research Project
163	1UH3NS103550-01	Electrophysiological Biomarkers To Optimize Dbs For Depression
164	1ZIAAG000342-01	Machine-Learning Approaches To Understand Preclinical Pathogenesis And Identify Predictive Biomarkers Of Alzheimers Disease
165	1ZIABC010837-11	Disease Prognostication And Treatment Response In Rectal Cancer Patients
166	1ZIACL040004-15	Computer Aided Detection For Radiologic Images
167	1ZIACL090018-08	Computer-Aided Detection For Infectious Disease Imaging
168	1ZIACT000199-28	Biomedical Imaging And Visualization
169	1ZIACT000271-15	Statistical Learning For Biomedical Data
170	1ZIACT000272-10	Biomedical Image Analysis And Informatics
171	1ZIADA000614-01	Mapping And Predicting Hiv-Transmission Hotspots With Phylogenetics And Geospatial Machine Learning
172	1ZIAHD008882-09	Functional And Structural Optical Brain Imaging
173	1ZIAHG000153-19	Development Of Statistical Genetics Methodology
174	1ZIALM010001-08	Integrating Image And Text Information For Biomedical Information Retrieval
175	1ZIALM010004-02	Image Analysis And Machine Learning For Pulmonary Disease Screening
176	1ZIALM010006-02	Malariascreener: Image Analysis And Machine Learning For Detecting Malaria In Blood Film
177	1ZIALM010009-01	Natural Language Processing For Precision Medicine And Clinical And Consumer Health Question
178	1ZIAMH002955-02	Non-Invasive Neuromodulation Unit (Nnu)
179	1ZIANS003055-10	Comprehensive Multimodal Analysis Of Patients With Neuroimmunological Diseases
180	1ZIASC010366-17	Identification Of Genes For Predicting Prognosis In Pediatric Cancers
181	1ZICHL006228-01	Bioformatics Core

182	1ZIHCT000200-28	Informatics; Machine Learning & Biomedical Data Science
183	1ZIHCT000260-22	Computational Tools For Bioinformatics And Genome Analysis
184	261201700515P-0-0-1	Igf::Ot::Igf Machine Learning Methods To Identify Bladder Cancer Cases Missed By Routine Surveillance
185	271201700022C-0-0-1	Igf::Ot::Igf Constructed Environments For Successfully Sustaining Abstinence Through Immersive And On-Demand Treatment. Period Of Performance: September 22; 2017 - March 21; 2018. N43da-17-5583.
186	2P01AG005842-30	4 - Assessing The Overuse And Underuse Of Diagnostic Testing
187	2P01AG005842-30	5 - The Role Of Information Technology & Treatment Heterogeneity In Population Health
188	2P01AG005842-30	Improving Health Outcomes For An Aging Population
189	2P30AG028740-11	Data Science And Applied Technology Core (Rc4)
190	2P30DK079312-11	Digital Analysis And Development
191	2P42ES004705-29	Core E: Data Science And Laboratory Core
192	2P50HD055784-11	Clinical Phenotyping Core
193	2P50HD055784-11	Genetics And Biomarkers Core
194	2R01DC011805-06	Imaging Genetics Of Spasmodic Dysphonia
195	2R01DC012033-06	Automated Measurement Of Language Outcomes For Neurodevelopmental Disorders
196	2R01EB006733-08	Development Of Robust Brain Measurement Tools Informed By Ultrahigh Field 7t Mri
197	2R01EB009352-09	The Xnat Imaging Informatics Platform
198	2R01EY023285-05	Functional And Structural Optical Coherence Tomography For Glaucoma
199	2R01GM105688-06	National Infrastructure For Standardized And Portable Ehr Phenotyping Algorithms
200	2R01GM108340-04	Methods; Tools And Resources For Interactive Online Virtual Screening And Lead Optimization
201	2R01LM011369-05	From Enrichment To Insights
202	2R01MH091864-06	Predicting Heterogeneous Neurodevelopmental Outcomes In School-Age Children With Early Caregiving Adversities
203	2R01MH096951-06	Automatic Multimodal Affect Detection For Research And Clinical Use
204	2R01NS073671-05A1	Statistical Methods For Early Disease Prediction And Treatment Strategy Estimation Using Biomarker Signatures
205	2R44AI124766-02A1	Long Non-Coding Rna Signatures To Classify Multiple Sclerosis
206	2R44MH104102-03	Tough Talks: A Disclosure Intervention For Hiv+ Ymsm
207	2R44TR000942-05	Biocomputation Across Distributed Private Datasets To Enhance Drug Discovery
208	2T32HL079896-11A1	Integrated Biostatistical Training For Cvd Research
209	2U01AA014809-14	Image Analysis Of Neurofacial Effects Of Prenatal Alcohol Exposure
210	2U19AI089676-08	Center For The Study Of Complex Malaria In India
211	2U54HG007480-05	Use Clinical; Genomic; And Immunological Data For Studying Viral And Host Biology
212	3F32GM116381-02S1	Tracing The Evolution Of The Human Mutation Rate
213	3F32MH108299-01A1S1	Neuroimaging And Machine Learning To Redefine Anxiety And Depression
214	3OT3TR002025-01S1	Biomedical Data Translator Technical Feasibility Assessment And Architecture Design

215	3OT3TR002026-01S1	Biomedical Data Translator Technical Feasibility Assessment And Architecture Design
216	3P30CA023108-38S4	Bioinformatics (Bisr)
217	3R01DC013547-04S1	Speech Movement Classification For Assessing And Treating Als
218	3R01EB006841-10S1	Multivariate Methods For Identifying Multitask/Multimodal Brain Imaging Biomarkers
219	3R01LM009254-11S1	Bio Text Nlp
220	3R01MH085651-08S1	Decision Processes Of Late-Life Suicide
221	3R43AG055203-01S1	Mobile Application To Deliver Personalized Nutrition For The Prevention Of Alzheimer's Disease
222	3T15LM007124-21S1	University Of Utah Biomedical Informatics Training Grant
223	3T15LM012500-01S1	An Interdisciplinary Program For Advanced Training In Health Data Analytics
224	3U01HG008679-03S1	Omop Information Model For Emerge Phenotyping
225	3U24DA041123-03S1	Abcd-Usa Consortium: Data Analysis Center
226	3U24DA041123-03S2	Abcd-Usa Consortium: Data Analysis Center
227	3U54EB020403-04S1	Enigma Center For Worldwide Medicine; Imaging & Genomics
228	3U54GM114838-03S2	Pilot For Creating Reproducible Workflows Using Docker Containers For Nih Commons
229	4R00EB020749-03	Stimulated Raman Imaging For Label-Free Histology To Guide Brain Tumor Surgery
230	5F30CA206291-02	Towards The Automation Of Mr Spectroscopic Imaging In Patients With Glioblashoma
231	5F30CA210329-02	Development Of A Multimodal Deep Learning Model For The Generation Of Cancer Probability Maps And Imaging Biomarkers For Prostate Cancer Using Multiparametric Mri
232	5F31CA210607-02	Discovery Of Pathogenic Germline Variants In Pediatric Cancers Using Genomics.
233	5F31DE025176-03	Signals Of Epigenetic Modification In Sjogrens Syndrome
234	5F31EY025532-03	Deciding Where To Look Next: Frontal Eye Field's Role During Natural Viewing
235	5F32GM116381-03	Tracing The Evolution Of The Human Mutation Rate
236	5F32NS093901-02	The Role Of Sleep In Insight And Generalization
237	5K01ES025442-04	Nonparametric Bayes Methods For Big Data In Neuroscience
238	5K01ES025445-03	Deep Learning And Streaming Analytics For Prediction Of Adverse Events In The Icu
239	5K01ES026832-02	Multi-Scale Data Integration Frameworks To Improve Cancer Outcomes
240	5K01ES026833-03	Multiparametric Prediction Of Vasospasm After Subarachnoid Hemorrhage
241	5K01ES026837-03	Data-Mining Clinical Decision Support From Electronic Health Records
242	5K01ES026839-03	Epileptic Biomarkers And Big Data: Identifying Brain Regions To Resect In Patients With Refractory Epilepsy
243	5K01HL124045-03	Novel Informatics Approaches For Ascertainment Of Pad Status And Adverse Outcomes
244	5K01LM012439-02	Managing Dementia Through A Multisensory Smart Phone Application To Support Aging In Place
245	5K01MH099141-05	Improved Methods To Assess The Comparative Safety Of New Psychiatric Medications
246	5K01MH102415-04	Application Of Cognitive; Emotional And Biological Dimensions - Resubmission - 1
247	5K01MH105625-03	Dynamic Brain Mechanisms Of Proactive And Reactive Control In Childhood Adhd

248	5K01MH106710-04	Developing Risk Algorithms Of Internalizing Disorder Etiology And Course
249	5K02AA023814-03	Using Technology To Scale Up The Evaluation Of Motivational Interviewing
250	5K02NS080885-05	Intrinsic Cortical Networks And Cognitive Dysfunction In Parkinson's Disease
251	5K02NS089852-04	Predicting Childhood Outcomes In Preterm Infants Using Cerebral Connectivity
252	5K07CA172677-05	Developing Smokers For Smoker (S4s): A Collective Intelligence Tailoring System
253	5K07CA211786-02	Improving Cancer Family History Collection Through Social Networking And Artificial Intelligence
254	5K07CA212057-02	Optimizing Long-Term Post-Polypectomy Surveillance For Colorectal Cancer Prevention Using A Prediction Rule Developed From A Large; Community-Based Cohort
255	5K08AG048321-04	Preventable Hospitalization In Dementia: The Impact Of Neuropsychiatric Symptoms
256	5K08HL121080-04	Predicting In-Hospital Cardiac Arrest Using Electronic Health Record Data
257	5K08MH107661-02	Evaluation Of Neurobiological Models Of Adolescent Maltreatment Through Machine Learning
258	5K23AG042856-05	Early Csf Detection Of Ftld
259	5K23AG045957-05	Early Detection Of Asymptomatic Middle-Age Adults At Risk For Ad
260	5K23DK101687-03	Improving Prediction Of Medical Responsiveness And Clinical Outcomes In Crohn's Disease
261	5K23HL119352-04	Investigating Sedentary Time In Aging: New Directions Using Technology (Istand)
262	5K23HL126912-03	Autoimmunity As A Mechanism For Atherosclerosis In Copd
263	5K23HL133495-02	Acute Respiratory Distress Syndrome After Isolated Traumatic Brain Injury: Platelet Biology; Endothelial Activation; And Mechanical Ventilation
264	5K23MH102128-04	Mhealth Intervention For Art Adherence And Sexual Risk Reduction Among Hiv+ Ymsm
265	5K24DA029262-08	Neuroimaging And Mentoring In Translational Pain Research
266	5K24NR015812-03	Mentoring And Research In Applying Digital Technologies To Prevent Chronic Illnesses
267	5K25CA181503-04	Quantitative Analysis Of Gbm Invasion Mechanisms With New Imaging Protocol
268	5K25DK097279-06	Understanding And Predicting Cardiac Events In Hd Using Real-Time Ehrs
269	5K25EB019032-03	Body Surface Tracking Of Complex Motion With Obstructed Viewing In Hybrid Imaging
270	5K25HL130637-02	Machine Learning Development For Subtyping Copd
271	5K99DA042127-02	Mechanisms Underlying Differential Effects Of Neighborhood Poverty On Problematic Adolescent Drug Use
272	5P01CA039542-29	Core 1: Administration And Biostatistics
273	5P01CA142538-08	Project 3: Statistical/Computational Methods For Pharmacogenomics And Individuali
274	5P01HL094307-07	Project 01: Mechanisms Of Extreme Phenotypes In Obstructive Sleep Apnea (Osa)
275	5P20GM103429-16	Bioinformatics Core
276	5P20GM113126-02	Data Management And Analysis Core
277	5P30AG021332-15	Biostatistics And Research Information Systems Core
278	5P30AG024832-13	Biostatistics & Data Management Resource Core 3
279	5P30AG044281-05	Pilot/Exploratory Studies
280	5P30CA023108-38	Bioinformatics (Bisr)

281	5P30CA030199-36	Bioinformatics
282	5P30CA036727-31	Bioinformatics (Bisr)
283	5P30CA042014-28	Research Informatics (Ri)
284	5P30CA042014-28	Utah Population Database (Updb)
285	5P30CA047904-29	Biostatistics Facility
286	5P30DA035778-04	Nida Center Of Excellence Of Computational Drug Abuse Research (Cdar)
287	5P30EY001319-43	Computing Module
288	5P30GM103324-05	Computational Resources Core (Crc)
289	5P30MH062294-15	Interdisciplinary Research Methods Core
290	5P41EB015909-17	Tr&D 3: Advanced Statistical Methods For Functional Mri
291	5P41GM103504-08	Tech R And D_Theme2
292	5P50AG033514-09	Data Management And Statistical Core
293	5P50CA097190-13	Core 3 Informatics; Biostatistics; And Bioinformatics
294	5P50CA121973-10	SPORE In Skin Cancer
295	5P50DC000422-29	Defining Phenotypes Of Age-Related Hearing Loss
296	5P50DC014664-02	Center For The Study Of Aphasia Recovery (C-STAR)
297	5P50GM107618-04	Administrative Core
298	5P50GM107618-04	Aim 4: Target Discovery For Common Disease Mechanisms
299	5P50GM107618-04	Education Core
300	5P50GM107618-04	Required Activities - Outreach
301	5P50GM107618-04	The HMS Laboratory Of Systems Pharmacology
302	5P50GM107618-04	Translational Pharmacology Core
303	5P50GM115305-04	Precision Phenomics To Personalize Drug Therapy (P3dt)
304	5P50GM115318-03	Informatics Core - Pharmacogenomics Of Statin Therapy (POST)
305	5P50MH090964-05	Statistical Models With High-Dimensional Predictors
306	5R00ES022986-05	Metal Mixtures; Children's Cognition; And Sensitive Developmental Windows
307	5R00LM011575-04	A Framework To Enhance Decision Support By Invoking Nlp: Methods And Applications
308	5R00LM011595-05	Novel Integrative Method To Detect Biomakers Of Breast Cancer Resistance
309	5R00LM012104-03	Natural Language Question Understanding For Electronic Health Records
310	5R01AA018673-07	Implementation Of Technology-Based Evaluation Of Motivational Interviewing
311	5R01AA024391-03	Dynamic; Real-Time Prediction Of Alcohol Use Lapse Using Mhealth Technologies
312	5R01AG021155-12	The Longitudinal Course O\F Imaging Biomarkers In People At Risk Of Ad
313	5R01AG041721-06	Quantifying Brain Abnormality By Multimodality Neuroimage Analysis
314	5R01AG042525-05	Metabolic Costs Of Daily Activities In Older Adults
315	5R01AG049371-03	Imaging Genomics Based Brain Disease Prediction
316	5R01AG053163-02	A Personalized Health Behavior System To Promote Well-Being In Older Adults
317	5R01AG054069-02	The Macrovascular And Microvascular Contributions To Alzheimer's Disease: Mesa Vascad
318	5R01AI116794-02	Biomedical Computing And Informatics Strategies For Infectious Disease Research
319	5R01AI117011-02	Tracking Evolution And Spread Of Viral Genomes By Geospatial Observation Error

320	5R01AI118833-03	Nasal Biomarkers Of Asthma
321	5R01AI127472-02	Enhanced Detection System For Healthcare-Associated Transmission Of Infection
322	5R01AR065479-04	Development And Dissemination Of Muscleminer: An Imaging Informatics Tool For Mus
323	5R01AT008448-04	Pain Care Quality And Integrated And Complementary Health Approaches
324	5R01AT008561-03	Single Session Pain Catastrophizing Treatment: Comparative Efficacy & Mechanisms
325	5R01CA158925-05	Integration Of Endoscopic And Ct Data For Radiation Therapy Treatment Planning
326	5R01CA172343-05	Digital Pathology_Accuracy Viewing Behavior And Image Characterization
327	5R01CA193730-02	Mri-Based Radiation Therapy Treatment Planning
328	5R01CA197000-02	Multi-Parametric 4-D Imaging Biomarkers For Neoadjuvant Treatment Response
329	5R01CA198121-02	Dose-Distribution Radiomics To Predict Morbidity Risk In Radiotherapy
330	5R01CA199673-03	Automated Image Guidance For Diagnosing Skin Cancer With Confocal Microscopy
331	5R01CA200690-02	Improving Melanoma Pathology Accuracy Through Computer Vision Techniques - The Impact Study
332	5R01CA200859-02	Hardwiring Mechanism Into Predicting Cancer Phenotypes By Computational Learning
333	5R01CA202752-02	Computerized Histologic Image Predictor Of Cancer Outcome
334	5R01CA203984-02	3-D Modeling-Based Decision Support For Optimizing Quality Of Life Following Breast Reconstruction
335	5R01CA204261-02	Integrated Discovery Pipeline For Tumor Neoantigens
336	5R01CA206100-02	Automatic Pelvic Organ Delineation In Prostate Cancer Treatment
337	5R01CA206180-02	Quantitative Multimodal Image Guidance For Improved Liver Cancer Treatment
338	5R01CA208517-02	Determinants Of Pancreatic Cancer And Malignant Melanoma Phenotypes In Cdkn2a Hereditary Kindreds
339	5R01CA214085-02	Sch: Exp: Improving Early Detection And Intervention Of Lymphedema
340	5R01DA035484-05	Fmri-Based Biomarkers For Multiple Components Of Pain
341	5R01DA039159-02	Reducing Non-Medical Opioid Use: An Automatically Adaptive Mhealth Intervention
342	5R01DA040487-03	Coinstac: Decentralized; Scalable Analysis Of Loosely Coupled Data
343	5R01DC004689-13	Therapeutic Approaches To Dysarthria: Acoustic And Perceptual Correlates
344	5R01DC007124-11	Dynamics Of Vocal Tract Shaping
345	5R01DC009834-08	Clinic Interactions Of A Brain-Computer Interface For Communication
346	5R01DC013547-04	Speech Movement Classification For Assessing And Treating Als
347	5R01DC014290-03	Using Machine Learning To Mitigate Reverberation Effects In Cochlear Implants
348	5R01DC014498-02	Computational Methods For The Study Of American Sign Language Nonmanuals Using Very Large Databases
349	5R01DK108073-04	Statistical Methods For Healthcare In Complex Patients With Diabetes
350	5R01EB001988-22	New Statistical Methods For Medical Signals And Images
351	5R01EB004640-11	Graph-Based Medical Image Segmentation In 3d And 4d
352	5R01EB006841-10	Multivariate Methods For Identifying Multitask/Multimodal Brain Imaging Biomarkers
353	5R01EB008374-07	4d Software Tools For Longitudinal Prediction Of Brain Disease
354	5R01EB016728-04	Optimization Of Mr Fingerprinting (Mrf) For Quantitative Mri
355	5R01EB019403-03	Secondary Use Of Emrs For Surgical Complication Surveillance

356	5R01EB021360-02	Machine Learning And Collaborative Filtering Tools For Personalized Education In Digital Breast Tomosynthesis
357	5R01EB021900-02	Sch: Int A.Soclotechnilcal Systems Systems Approach For Improving Tuberculosis Diagnostics Using Mobile Health Technologies
358	5R01EB022573-02	Pattern Analysis Of Fmri Via Machine Learning/Sparse Models: Application To Brain Development
359	5R01EB022880-02	Diagnosis Of Alzheimer's Disease Using Dynamic High-Order Brain Networks
360	5R01EB022883-02	Manifold-Valued Statistical Models For Longitudinal Morphometic Analysis In Preclinical Alzheimer's Disease (Ad)
361	5R01ES026033-02	Novel Biomarker To Identify Critical Windows Of Susceptibility To Metal Mixture
362	5R01EY023279-05	3d Image Analysis Approach To Determine Severity And Cause Of Optic Nerve Edema
363	5R01EY026593-02	Developing Classification Criteria For The Uveitides
364	5R01GM071966-12	Integration And Visualization Of Diverse Biological Data
365	5R01GM088224-07	Real-Time Detection Of Deviations In Clinical Care In Icu Data Streams
366	5R01GM097618-06	Machine Learning For Identifying Adverse Drug Events
367	5R01GM101430-05	Mining Health Data For Drug Safety Profiles
368	5R01GM108731-03	A Three-Population Three-Scale Social Network Model To Assess Disease Dispersion
369	5R01GM111324-03	Comparative Assessment Framework For Environments Of Trauma Care
370	5R01GM114290-03	Finding Good Temporal Postoperative Pain Signatures (Tempos)
371	5R01GM114355-02	Extended Methods And Software Development For Health Nlp
372	5R01GM114434-03	Boosting The Translational Impact Of Scientific Competitions By Ensemble Learning
373	5R01GM117622-02	Machine Learning Of Physiological Variables To Predict Diagnose And Treat Cardiorespiratory Instability
374	5R01GM122062-02	Transmission Networks In Trait-Based Communities
375	5R01HD076756-05	Profiling Vulvodynia Based On Neurobiological And Behavioral Endophenotypes
376	5R01HD083431-02	Novel Approaches For Predicting Unstructured Short Periods Of Physical Activities In Youth
377	5R01HD084628-03	Misfoldome-Centered Multiomics Approach To Unravel Preeclampsia Subphenotypes.
378	5R01HD087133-03	Sch: Gear - Grounded Early Adaptive Rehabilitation
379	5R01HL089765-09	High Performance Automated System For Analysis Of Fast Cardiac Spect
380	5R01HL116522-05	An Evaluation Of Novel Domains For Predicting 30-Day Readmission
381	5R01HL121226-04	Integrated Rf And B-Mode Deformation Analysis For 4d Stress Echocardiography
382	5R01HL122010-04	Decrypting Variants Of Uncertain Significance In Long-Qt Syndrome
383	5R01HL122484-04	Probing Dose Limits In Cardiac Spect With Reconstruction And Personalized Imaging
384	5R01HL122639-03	Cora_Tm_A Personalized Cardiac Counselor For Optimal Therapy
385	5R01HL125089-03	Ehr Anticoagulants Pharmacovigilance
386	5R01HL125583-03	Integrative Genomics Of Clinical Subtypes In Copdgene
387	5R01HL126667-03	Asthma Ascertainment And Characterization Through Electronic Health Records
388	5R01HL127028-03	Transition From Risk Factors To Early Hf: Prevalence; Pathogenesis; And Phenomics
389	5R01HL127661-03	Innovative Mri-Based Characterization Of Cardiac Dyssynchrony

390	5R01HL133616-02	Automated Quantitative Ct Imaging Of Epicardial Adipose Tissue And Risk Of Cardiac Events
391	5R01LM008111-12	Developing And Applying Information Extraction Resources And Technology To Create
392	5R01LM009254-11	Bio Text Nlp
393	5R01LM010090-07	Temporal Relation Discovery For Clinical Text
394	5R01LM010207-09	Automated Detection Of Anomalous Accesses To Electronic Health Records
395	5R01LM010681-08	Interactive Machine Learning Methods For Clinical Natural Language Processing
396	5R01LM010685-08	From Gwas To Phewas: Scanning The Emr Phenome For Gene-Disease Associations
397	5R01LM010817-06	Text Mining Pipeline To Accelerate Systematic Reviews In Evidence-Based Medicine
398	5R01LM011663-04	A New Generation Clinical Decision Support System
399	5R01LM011829-04	Patient Medical History Representation; Extraction; And Inference From Ehr Data
400	5R01LM011934-04	Semi-Structured Information Retrieval In Clinical Text For Cohort Identification
401	5R01LM011975-03	Evidence-Based Strategy And Tool To Simplify Text For Patients And Consumers
402	5R01LM012012-03	Bioinformatics Strategies To Relate Age Of Onset With Gene-Gene Interaction
403	5R01LM012086-03	Semi-Automating Data Extraction For Systematic Reviews
404	5R01LM012095-03	Development And Evaluation Of A Learning Electronic Medical Record System
405	5R01LM012309-02	Predicting Diabetic Retinopathy From Risk Factor Data And Digital Retinal Images
406	5R01LM012355-03	The Next Frontier In Diabetes Communication: Promoting Health Literacy In The Era Of Secure Messaging
407	5R01MH059929-17	Course And Outcome Of Bipolar Disorder In Youth
408	5R01MH085651-08	Decision Processes Of Late-Life Suicide
409	5R01MH096773-06	Characterizing Mechanistic Heterogeneity Across Adhd And Autism
410	5R01MH100482-05	Artificial Intelligence In A Mobile Intervention For Depression (Aim)
411	5R01MH103539-03	Treatment Utilization Before Suicide (Tubs)
412	5R01MH104414-04	Connectomics In Psychiatric Classification
413	5R01MH105355-03	Neural Signature Of Fear Overgeneralization In Trauma Exposed Adults
414	5R01MH106577-02	Natural Language Processing For Characterizing Psychopathology
415	5R01MH107558-02	Thought Disorder And Social Cognition In Clinical Risk States For Schizophrenia
416	5R01MH107797-03	Inside The Social Perception Network: Dynamics; Connectivity; And Stimulation
417	5R01MH109320-03	Dot Diary (D2): Developing A Mobile App With Combined Automated Dot And Daily Sexual Diary For Monitoring And Improving Prep Adherence
418	5R01MH109329-02	Socioemotional Processing In Female Offenders - Resubmission 01
419	5R01MH109496-02	Novel Methods For Evaluation And Implementation Of Behavioral Intervention Technologies For Depression
420	5R01MH109507-02	Risk Profiles For Suicidal Behavior In The General Population
421	5R01MH110483-02	Study Of Early Brain Alterations That Predict Development Of Chronic Ptsd
422	5R01MH110514-02	Behavioral; Physiological; And Quantitative Models Of Pro-Social Behavior
423	5R01NR013912-05	Predicting Patient Instability Noninvasively For Nursing Care-Two (Ppinnc-2)
424	5R01NS042645-14	Predicting Brain Tumor Progression Via Multiparametric Image Analysis And Modelin
425	5R01NS047293-13	Eeglab: Software For Analysis Of Human Brain Dynamics

426	5R01NS049251-11	Multimodal Registration Of The Brain's Cortical Surface
427	5R01NS086885-04	White Matter Damage In Subconcussive Blast Exposure
428	5R01NS088606-04	Multi-Scale Network Dynamics Of Human Upper Limb Movements: Characterization And Translation To Neuroprosthetics
429	5R01NS089212-03	A Brain Atlas For Mapping Connectivity In Focal Epilepsy
430	5R01NS089729-04	Neural Mechanisms For Reducing Interference During Episodic Memory Formation
431	5R01NS092882-03	Reliable Seizure Prediction Using Physiological Signals And Machine Learning
432	5R01NS094399-03	Characterizing High Frequency Oscillations As An Epilepsy Biomarker With Big Data Tools
433	5R01NS095741-03	Stroke; Brain Networks; And Behavior
434	5R01NS097000-02	Whole Transcriptome Studies Of Patients With Transient Ischemic Attacks (Tias)
435	5R01NS098023-02	Integrating Ehr And Genomics To Predict Multiple Sclerosis Drug Response
436	5R03DA040855-02	Automated Speech Analysis: A Marker Of Drug Intoxication & Treatment Outcome
437	5R03ES026397-02	Development Of Integrative Models For Early Liver Toxicity Assessment
438	5R03MH108933-02	Automated Linguistic Analyses Of Semantics And Syntax In Speech Output In The Psychosis Prodrome: A Novel Paradigm To Evaluate Subtle Thought Disorder.
439	5R03MH109008-02	Separating Wheat From Chaff In Major Depression Blood Biomarker Studies
440	5R03MH109791-02	Neuroanatomical Markers Of Persistence Versus Remission Of Adhd
441	5R21AA024530-02	Mediators And Moderators Of A Neighborhood Experiment On Alcohol Use
442	5R21AG050122-02	Multi-Modal Prediction Of Future Clinical Dementia
443	5R21AG053467-02	Computational Modeling Of Semantic Decline In Alzheimer's Disease
444	5R21CA194492-02	Automated Ecological Video Identification Of Physical Activity (E-Vip) Software
445	5R21CA202130-02	Deep Learning For Representation Of Codes Used For Seer-Medicare Claims Research
446	5R21CA209874-02	Developing Enabling Pet-Ct Image Analysis Tools For Predicting Response In Radiation Cancer Therapy
447	5R21DK108071-02	Automated Coding Of Ecoaching Exchanges To Promote Healthier Eating
448	5R21DK108104-02	Promoting Utilization Of Kidneys By Improving Patient Level Decision Making
449	5R21EB022356-02	Adaptive Prediction Of Blood Glucose Levels Using Wearable Physiological Sensors
450	5R21EB022747-02	Deep-Radiomics-Learning For Mass Detection In Ct Colonography
451	5R21HD086745-02	Machine Learning Algorithms To Measure Physical Activity In Children With Cerebral Palsy
452	5R21HD086754-02	Childhood Obesity Surveillance Using Electronic Health Records Data
453	5R21HL131385-02	Simultaneous Imaging Of Myocardial Blood Flow And Glucose Metabolism Using Dynamic 18f-Fdg Pet
454	5R21HL132277-02	Computerized Visualization And Prediction Of Coronary Artery Ischemia
455	5R21HL133891-02	Endotypes Of Thrombocytopenia In The Critically Ill
456	5R21MH108999-02	Building Multistage Treatment Regimens For Depression After Acute Coronary Syndromesyndrome
457	5R21MH110758-02	Machine Learning And Personalized Prognosis For Depression Treatment
458	5R21NR015410-02	Activity-Aware Prompting To Improve Medication Adherence In Heart Failure Patients
459	5R21NS090349-02	Pattern Classification Using Magnetic Resonance Imaging In Traumatic Brain Injury

460	5R21NS093266-02	Characterizing Intrinsic Functional Cortical Networks In Parkinson Disease Dementia
461	5R21TW010245-02	Low Cost Mobile Platform For Pulmonary Disease Screening
462	5R24DK106766-02	Vision: Validated Systematic Integration Of Epigenomic Data
463	5R25EB022363-03	Transforming Analytical Learning In The Era Of Big Data
464	5R25EB022366-03	The Big Dipa: Data Image Processing And Analysis
465	5R33CA206922-02	Online Raman Diagnostics Of Oncometabolites
466	5R33MH100268-04	Development Of A Novel Neurotechnology To Promote Emotion Recognition In Autism
467	5R34MH110583-02	Scaling A Smarter And More Efficient Intervention: Evaluating The Feasibility Of Disseminating A Novel Mobile App Platform To Treat Depression
468	5R35GM119582-02	Statistical Methods For Real-Time Forecasts Of Infectious Disease: Dynamic Time-Series And Machine Learning Approaches
469	5R42AG055375-04	Development Of An Integrated High Throughput Imaging And Image Analysis Platform For Muscle
470	5R42CA180190-03	Automated Problem And Allergy Lists Enrichment Based On High Accuracy Information Extraction From The Electronic Health Record
471	5R44AG050326-02	Integrated Neurocognitive And Sleep-Behavior Profiler For The Endophenotypic Classification Of Dementia Subtypes (Inspecds)
472	5R44EY026864-06	Advanced Image Analysis Tools For Diabetic Retinopathy Telemedicine Application
473	5R44HL125001-03	Corband: A Novel Wearable Wrist Sensor For Heart Failure Remote Monitoring
474	5R44NS089090-03	Mri Brain Morphometry For Computer-Aided Detection Of Neurological Disorders
475	5R44NS092209-03	Advanced Morphological Analysis Of Cerebral Blood Flow For Acute Concussion Diagnosis And Return-To-Play Determination
476	5R90DA043849-02	Training A New Generation Of Computational Neuroscientists Bridging Neurobiology
477	5SC2GM118266-02	A Novel Probabilistic Methodology For Prediction Of Emerging Diseases In Patients With Multiple Chronic Conditions
478	5T32HG002295-15	Training In Bioinformatics And Integrative Genomics
479	5T32LM012409-02	Biomedical Data Science Graduate Training At Stanford
480	5T32LM012417-02	Biomedical Big Data Training Program At Uc Berkeley
481	5T32LM012419-03	University Of Washington Phd Training In Big Data For Genomics And Neuroscience
482	5T32NS091006-03	Training Program In Neuroengineering And Medicine
483	5T90DA022762-12	Interdisciplinary Training In Computational Neuroscience
484	5T90DA043219-02	Training A New Generation Of Computational Neuroscientists Bridging Neurobiology And Cognition
485	5U01CA179106-04	Biomarkers For Staging And Treatment Response Monitoring Of Bladder Cancer
486	5U01CA187947-03	Computing; Optimizing; And Evaluating Quantitative Cancer Imaging Biomarkers
487	5U01CA195599-03	Quantitative Parenchyma Descriptor As An Imaging Biomarker Of Breast Cancer Risk
488	5U01DK065184-15	Michigan Hepatotoxicity Clinical Research Network Renewal 2013
489	5U01DK103149-04	Clinical Center For Children: Pathogenesis; Biomarkers; And Antifibrotic Therapy
490	5U01HG008390-03	Mace2k - Molecular And Clinical Extraction: A Natural Language Processing Tool For Personalized Medicine
491	5U01HG008488-03	Mining The Social Web To Monitor Public Health And Hiv Risk Behaviors

492	5U01HG009454-02	Metadata Applications On Informed Content To Facilitate Biorepository Data Regulation And Sharing
493	5U01MH110925-02	Longitudinal Assessment Of Post-Traumatic Syndromes
494	5U19AI109713-04	Bayesian Models To Accelerate Antibacterial Drug Discovery
495	5U24CA184407-04	Cancer Deep Phenotype Extraction From Electronic Medical Records
496	5U24CA194215-02	Advancing Cancer Pharmacoepidemiology Research Through Ehrs And Informatics
497	5U24CA210952-02	Integrative Mirna Data Analysis For Clinical Cancer Genomics
498	5U24CA210972-02	Proteogenomic Data Analysis For Cancer Systems Biology And Clinical Translation
499	5U24CA210990-02	Ucsc-Buck Specialized Genomic Data Analysis Center For The Genomic Data Analysis Network
500	5U54AI117924-04	Administration
501	5U54AI117924-04	Bd2k Center Consortium
502	5U54AI117924-04	Data Science Research
503	5U54AI117924-04	Training
504	5U54EB020403-04	Data Science Research
505	5U54EB020403-04	ENIGMA Center For Worldwide Medicine; Imaging & Genomics
506	5U54EB020405-04	Data Science Research Core
507	5U54HD090256-02	Impact Of Cgg Repeats On Fmr1 Gene Function And Human Health
508	5U54HG008098-04	Data Analysis And Signature Generation Core
509	5U54MD010706-02	Data Integration Core
510	5U54MD010722-02	Bio Repository Core
511	5UH2CA203708-02	Crowd Sourcing Labels From Electronic Medical Records To Enable Biomedical Research
512	5UH2CA203711-02	Crowdsourcing Mark-Up Of The Medical Literature To Support Evidence-Based Medicine And Develop Automated Annotation Capabilities
513	5UH2CA203730-02	Crowdsourcing-Aided Machine Learning For Colon Cancer Prevention
514	5UH2EB024407-02	Crowd-Sourced Annotation Of Longitudinal Sensor Data To Enhance Data-Driven Precision Medicine For Behavioral Health
515	5UH2NS100599-02	Multimodal Mri Biomarkers Of Small Vessel Disease For Older Persons With And Without Dementia.
516	7R01CA197398-03	Computational Model Of Autophagy-Mediated Survival In Chemoresistant Lung Cancer
517	7R01DC011805-07	Imaging Genetics Of Spasmodic Dysphonia
518	7R01MD010362-03	Eliminating Tobacco-Related Disparities Amount African American Smokers
519	7R01MH108728-02	Understanding The Functional Impacts Of Genetic Variants In Mental Disorders
520	7U24CA184407-05	Cancer Deep Phenotype Extraction From Electronic Medical Records
521	9R01GM125072-05A1	Nextgen Random Forests
522	1R01NS101718-01A1	Whole Genome RNA Sequencing (Rnaseq) Of Blood From Patients With Lacunar Stroke
523	1R21LM012578-01	Implementing And Evaluating A Machine Learning Tool For Entity Resolution In Drug Use And Sexual Contact Networks Of YMSM
524	1R21MH114835-01	Mhealth Monitoring Of Acoustic And Behavioral Patterns In Bipolar Disorder Across Cultures

525	1UG3AT009758-01	Engaging Veterans Seeking Service-Connection Payments In Pain Treatment
526	2P50HD052120-11	Project III: Understanding Risk For Early Language And Literacy Difficulties In Young Children
527	2T15LM007033-34	Biomedical Informatics Training At Stanford
528	2T15LM007124-21	University Of Utah Biomedical Informatics Training Grant
529	3R01EB021396-02S1	Slicer+PLUS: Collaborative; Open-Source Software For Ultrasound Analysis
530	5R01EB021396-02	Slicer+PLUS: Collaborative; Open-Source Software For Ultrasound Analysis
531	5R01NR015371-03	NRI: An Egocentric Computer Vision Based Active Learning Co-Robot Wheelchair
532	5R01NR016151-03	Nri: Collaborative Research: Quadrupedal Human-Assistive Robotic Platform (Q-Harp)
533	5U01DA041089-03	Abcd-Usa Consortium: Research Project
534	5U54AI117924-04	The Center For Predictive Computational Phenotyping-1 Overall
535	5U54GM114833-04	A Community Effort To Translate Protein Data To Knowledge: An Integrated Platform
